# Space Charge Characteristics at the Interface of Laminated Epoxy Resin

**DOI:** 10.3390/molecules28145537

**Published:** 2023-07-20

**Authors:** Yifan Zhang, Bing Luo, Mingli Fu, Lei Jia, Chi Chen, Gang Zhou, Chuang Wang

**Affiliations:** 1 Electric Power Research Institute, China Southern Power Grid, Guangzhou 510663, China; luobing@csg.cn (B.L.); fuml@csg.cn (M.F.); jialei@scg.cn (L.J.); 2 National Engineering Research Center for UHV Power Technology and New Power Equipment, Guangzhou 510663, China; 3 School of Electrical Engineering, Xi’an University of Technology, Xi’an 710048, China; chenchi0129@stu.xjtu.edu.cn (C.C.); chenhandagege@163.com (G.Z.)

**Keywords:** TSDC, epoxy resin, space charge, interlayer interface

## Abstract

In the design and manufacturing of epoxy resin insulation components, complex structures can be achieved through multiple pours, thereby forming the structure of interface of laminated epoxy resin. This type of interface structure is often considered a weak link in performance which can easily accumulate charges and cause electric field distortion. However, research on the interlayer interface of epoxy resin has received little attention. In this study, epoxy samples with and without interlayer interfaces were prepared, and the space charge accumulation characteristics and trap characteristics of the samples were analyzed via pulsed electro-acoustic (PEA) and thermally stimulated depolarization current (TSDC) methods. The experimental results indicate that the Maxwell–Wagner interface polarization model cannot fully explain the charge accumulation at the interface. Due to the influence of the secondary curing, the functional groups in the post-curing epoxy resin can move and react with the partially reacted functional groups in the prefabricated epoxy resin layer, resulting in a weak cross-linking network at the interface. With the increase in temperature, the molecular chain segments in the weak cross-linked region of the interface become more active and introduce deep traps at the interface, thereby exacerbating the accumulation of interface charges. In addition, due to the influence of interface polarization and weak cross-linking, the ability of the interface charges to cause field strength distortions decreases with the increase in applied field strength. This research study can provide a theoretical reference for the interfacial space charge transport characteristics of epoxy-cured cross-linked layers and provide ideas for regulating interfacial cross-linking to suppress interfacial charge accumulation.

## 1. Introduction

Epoxy resin is an excellent solid insulation material with excellent molding and processing properties [1,2,3]. The interface structure formed between two layers of epoxy resin may appear in large and complex epoxy casting parts due to process or design reasons and is generated by multiple castings of epoxy resin. For example, the basin insulators used in gas-insulated switchgears (GIS) may have the interface of laminated epoxy resin between the epoxy resin interface buffer material and the basin epoxy insulation material, as well as the interface formed by multi-layer and multiple castings in large dry-type transformers and dry-type bushings [4,5,6,7,8]. The interlayer interface formed by multi-layer epoxy resin is different from the contact interface formed by general solid insulation materials, and there is a cross-linking and curing process of epoxy resin during its formation. The high electric field and high-temperature operating environments of power equipment can cause interlayer interfaces to work under more severe conditions, resulting in an easier accumulation of space charges at the interface [9,10,11]. The accumulated space charges tend to distort the local electric field, and in serious cases, will cause electrical breakdown damage from the interface so that the interlayer interface becomes the weak link of the entire insulation system.

Das S et al. measured the interfacial charges at the discontinuous surfaces of epoxy resin and polyethylene polymer composite materials, and the results showed that for epoxy polyethylene composite materials, interfacial charges would accumulate at the interface due to the differences in dielectric constant and conductivity between the two materials. As the electric field intensity increases, the positive polarity charge is trapped in the interface area [12]. Wen Cao et al. [13] studied the space charge properties of epoxy resin/insulating paper composites. The research showed that space charges tend to accumulate at the epoxy/paper interface under low electric field conditions in epoxy/paper composites, and the amount of interface charge decreases with the increase in voltage, resulting in a more uniform field distribution in the composite material. This behavior led to different breakdown properties between pure epoxy resin and epoxy resin/paper composite materials.

The space charge characteristics at the interface of double-layer insulating media are mostly based on the Maxwell–Wagner polarization model, but due to the complexity of the actual physical interface, the model also has analytical defects. The Maxwell–Wagner effect is characterized by the accumulation or redistribution of charges at the interface. It arises from a difference in conductivity or permittivity between the two materials. When an electric field is applied, charges tend to accumulate near the electrodes. In addition to charge accumulation, the Maxwell–Wagner effect is also associated with polarization. When an electric field is applied, the dielectric material undergoes polarization, causing the alignment of dipoles or charge separation within the material. And the accumulated charges at the interface create an electric field that affects the charge distribution within the composite material. This leads to further charge redistribution and polarization effects within the dielectric material. Lan et al. [14] tested and analyzed the space charge distribution of a low-density polyethylene/Ethylene propylene rubber (LDPE-EPR) double-layer medium and found that the interface space charge of the double-layer medium was composed of a polarization charge caused by polarization and a cathode-injected electron charge. The team also studied the accumulation of negative interfacial charges due to the capture of deep traps on the surface of ethylene propylene rubber during the migration of cathode-injected electrons into the anode, which caused the results to not meet the polarization model. Wu Kai et al. [15] demonstrated the complexity of interface charge behavior through simulation and measurement results, and they found that interface charges in multi-layer insulation structures may not follow the Maxwell–Wagner model, and interface charge behavior is related to contact type and external stress. At present, a large number of interfaces formed by pressure contact have been studied, and the interfacial structures formed by curing and cross-linking are less studied; in particular, the mechanisms of the effects of curing and cross-linking reactions on the space charge accumulation properties in the interface of laminated epoxy resin must be explored.

In this study, epoxy samples with and without interlayer interfaces were prepared, and the space charge accumulation and trap characteristics of the samples were measured and analyzed via pulsed electro-acoustic (PEA) and thermally stimulated depolarization current (TSDC) methods. Through these analyses, the main factors affecting the interface charge were studied, and the influence of the interface structure on electrical performance and its mechanism were discussed. The above research may provide a theoretical reference for the interfacial space charge transport characteristics of epoxy-cured cross-linked layers and provide ideas for regulating interfacial cross-linking to suppress interfacial charge accumulation.

## 2. Experimental Result

### 2.1. PEA

**Figure 1:**
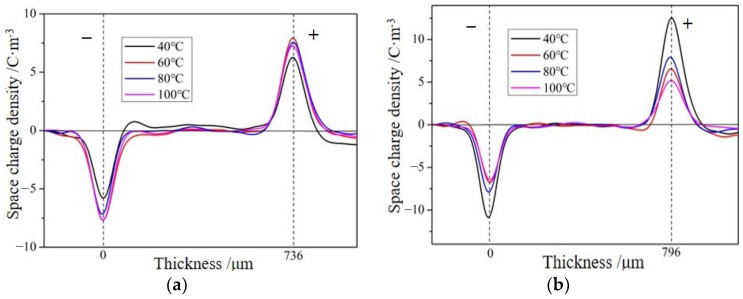
Space charge distribution in samples without interfaces at different temperatures and field strengths. (**a**) Result of applying a field strength of 20 kV/mm to a portion of the sample during one pouring. (**b**) Result of applying a field strength of 20 kV/mm to the sample during secondary pouring.

From Section 4.1, it can be seen that the two layers of epoxy resin in this article were formed by curing and cross-linking. Unlike the crimping structural material, there is no air gap at the interface, so the double-layer epoxy resin material in this article can be considered to have perfect contact. The analyses of interface microstructure in previous studies on interfaces between two layers of epoxy resins also indicate that the interface here is not a simple contact interface [16]. The two layers of epoxy resin use the same material, but the post-cast layer undergoes two curing processes, which is equivalent to adding an “aging” process on top of adding prefabricated epoxy samples. It is necessary to consider whether this process will affect the spatial charge distribution within the material. Therefore, the space charges inside the interface were measured at different temperatures and field strengths for three types of samples with and without interfaces, as shown in Figure 1. However, considering the overshoot temperature difference in experimental testing, there is no obvious charge accumulation phenomenon inside the first and second pouring parts of the samples without interfaces at different temperatures and field strengths. This is because the injection potential barrier between the epoxy resin formula and the electrode interface is high at this temperature and field strength, so there is no obvious charge injection inside the epoxy resin body, Therefore, the impact of pouring twice on the spatial charge distribution of the material will no longer be considered in the following text.

**Figure 2:**
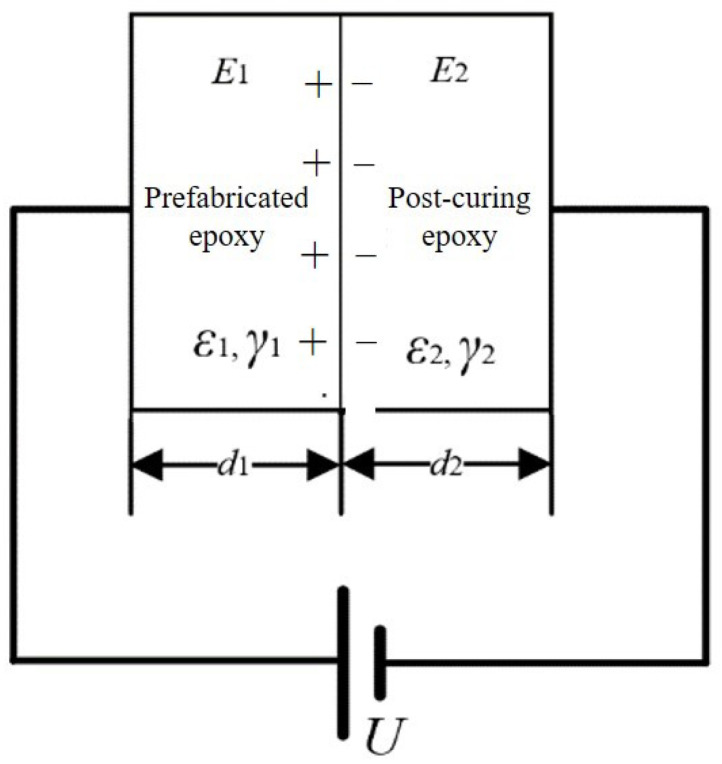
Circuit diagram of double-layer dielectric under an external electric field.

When there is an interface formed by solidification in a material, it is equivalent to a double-layer dielectric. Although the formulation system and curing process of the epoxy resin on both sides of the interface are the same, due to the fact that the material in the second pouring part of the interface sample undergoes one curing process and the first pouring part undergoes two curing processes, there are small differences in the dielectric constants and conductivities of the epoxy resins on either side of the interface. Under an applied voltage, interface polarization may occur at the interface, and charges may appear at the interface. When voltage is applied on both sides of the material to form a circuit, the circuit is shown in Figure 2 [17].

When a voltage is applied at both ends of the material and the voltage is small enough to not cause the field strength at both ends of the material to exceed the injection threshold, only interface polarization exists at the interface, and the expression for the charge density at the interface is shown in Formula 1. The polarity of the interfacial charge is consistent with the polarity of the electrode applied on the left side of the secondary casting part and is positive.

(1) $$\sigma =\frac{\varepsilon_{2}\gamma_{1}-\varepsilon_{1}\gamma_{2}}{\gamma_{1}d_{2}+\gamma_{2}d_{1}}U$$

where *ε*_1_ and *ε*_2_ are relative dielectric constants of the prefabricated epoxy and post-curing epoxy; *γ*_1_ and *γ*_2_ are the electrical conductivity values of the prefabricated epoxy and post-curing epoxy.

**Figure 3:**
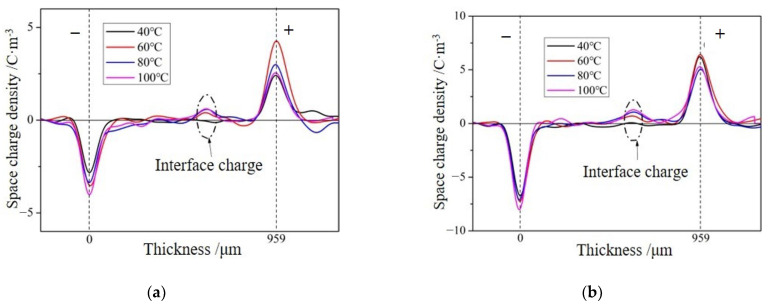
Space charge distribution in samples with interfaces at different temperatures and field strengths. (**a**) Results of applying a field strength of 10 kV/mm to samples containing interfaces. (**b**) Result of applying field strength of 20 kV/mm to the sample with an interface.

**Figure 4:**
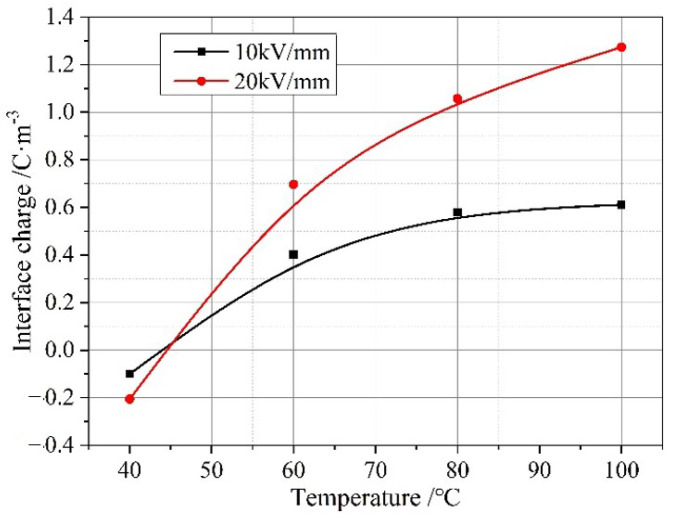
Space charge results of interface samples under pressure for one hour at different temperatures and field strengths.

From Figure 3 and Figure 4, it can be seen that with increases in field strength and temperature, the accumulated charges at the interface gradually increase. At low temperatures and low fields, there is almost no space charge at the interface. There may be two main reasons for the generation of interface charges here. On the one hand, there are slight differences in the conductivity and relative dielectric constant between the epoxy resins on either side of the interface. At low temperatures and low fields, this difference is very small, resulting in no obvious interface charge appearing at the interface. As the temperature increases, there is a very small difference in the relative dielectric constant between samples with and without interfaces within the temperature range of 40 °C to 100 °C, while the conductivity of epoxy resin increases linearly in logarithmic coordinates [18]. Therefore, the numerical difference between the conductivities gradually increases, leading to an increase in the space charge at the interface. Similarly, when the conductivity is less than the percolation threshold, it shows an almost linear increase with the increase in field strength in the logarithmic coordinate system, which also increases the numerical difference between the conductivities and leads to an increase in the space charge at the interface.

**Figure 5:**
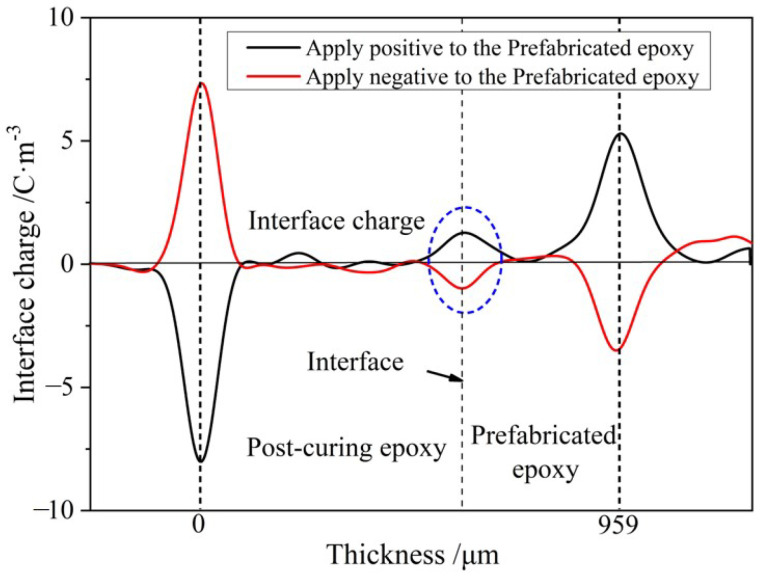
Space charge distribution at the interface after changing the positive and negative electrodes of the electrode.

According to the model of interface polarization, if the charge on the interface is caused by the difference in material properties on both sides of the interface, then the polarity of the accumulated charge at the interface will also change by changing the positive and negative electrodes in the experiment. In order to verify the type of interface charge, the positive and negative electrodes were changed, and the space charge distribution of the sample containing the interface was measured at 100 °C and 20 kV/mm and compared with the results in Figure 5. The internal space charge distribution was obtained.

**Figure 6:**
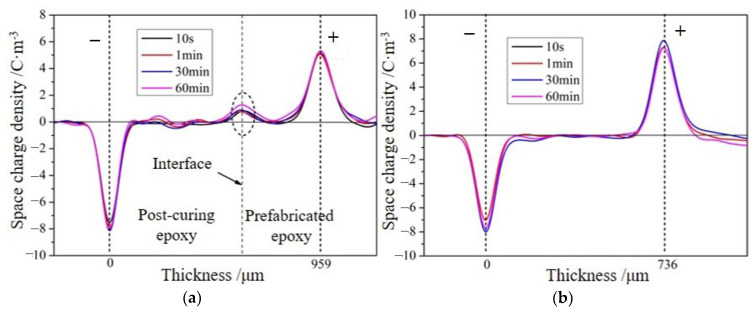
Space charges of samples with and without interfaces at different pressurization times (20 kV/mm, 100 °C); (**a**) interface specimens; (**b**) interface-free specimens.

From Figure 5, it can be seen that after changing the polarity of the electrodes on both sides of the sample, the polarity of the charges at the interface changes, which is consistent with the interface polarization model, indicating that the charges accumulated at the interface mainly come from interface polarization. According to the results of measuring the dielectric properties, the relative dielectric constants of the primary casting part and the secondary casting part are close, while the conductivity of the primary casting part is larger than that of the secondary casting part, so it is consistent with *ε*_1_*γ*_2_ > *ε*_2_*γ*_1_, and the charge polarity at the interface is the same as the loading voltage polarity of the primary casting part. When the right side of the specimen is loaded as the anode, the charge density at the interface is 1.27 C/m^3^; when the right side of the specimen is loaded as the cathode, the charge density at the interface is −0.98 C/m^3^. Since the amounts of charge generated by interfacial polarization should be equal, the difference between the two indicates that there are other factors affecting the accumulation of space charges at the interface. When the applied field strength was 20 kV/mm, the formation of space charges during the process of pressurizing the sample at 100 °C was measured, and the distribution of space charges within the sample at different pressurization times is shown in Figure 6. During the pressurization process, there was no interfacial charge observed in the interface-free sample. For the interface samples, interface charges appeared on the interface after the voltage stabilizes, and the amount of interface charges remained basically unchanged before 30 min of pressurization. However, the amount of interface charges increased again after 60 min of pressurization. The charge formed in the initial stage should be caused by interface polarization. When the voltage stabilizes, interface polarization already occurs and charges are formed at the interface. As the pressurization time increases, some traps at the interface capture charge carriers, causing a small increase in charge at the interface once again.

**Figure 7:**
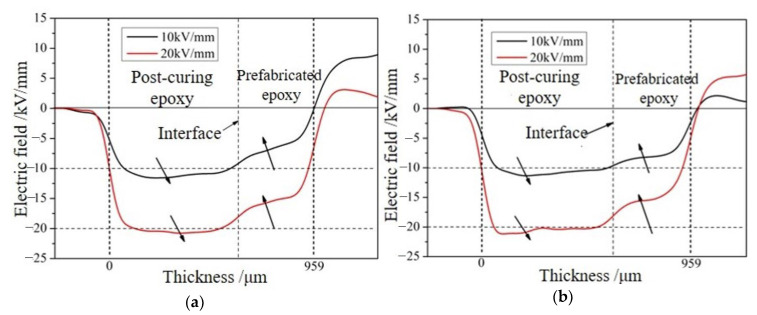
Effect of the boundary surface on the electric field distribution in the sample at different temperatures and field strengths. (**a**) Electric field distribution under different field strengths at 80 °C. (**b**) Electric field distribution under different field strengths at 100 °C.

The presence of space charges affects the distribution of the electric field in the sample, causing distortion. On the basis of measuring the distribution of space charges in the sample, the electric field distribution in the sample was calculated at different temperatures and field strengths after 1 h of pressurization. The electric field distribution in the sample under different field strengths was obtained as shown in Figure 7.

When there is no interface present, the applied electric field is consistent with the internal electric field of the material. For samples containing interfaces, due to the synergistic effect of polarization and weak cross-linking at the interface, the electric field distribution in the sample is distorted. The polarity of the charged electrode at the interface is the same as the polarity of the loading electrode on the right side of the sample, so the same type of charge accumulates on both sides of the pouring section, resulting in a decrease in the electric field strength and voltage in this area. The charge accumulated at the interface is different from the polarity of the loading electrode on the left side of the sample, which is equivalent to narrowing the distance between the positive and negative electrodes, causing this part to bear an increase in voltage and electric field strength. The field strength distortion factor *F*_d_ is defined as shown in Formula 2:

(2) $$F_{d}=\frac{E_{\max}-E_{p}}{E_{p}}\times 100\%$$

where *E*_max_ and *E*_p_ are the maximum field strength inside the sample and the average applied field strength, respectively. The maximum field strength and its distortion factor calculated, at different temperatures, are listed in Table 1.

The field strength distortion decreases with the increase in the applied field strength, and the temperature has little effect on the field strength distortion. The number of accumulated charges at the interface increases with the increase in applied field strength from 10 kV/mm to 20 kV/mm, but the increase is less than double, which leads to a decrease in the ability of the interface charges to cause field strength distortion with the increase in the applied field strength. This is because as the temperature increases, the degree of weak cross-linking at the interface strengthens, and deeper level traps are introduced at the interface. Deep traps reduce the mobility of charge carriers, resulting in the above phenomenon.

### 2.2. TSDC

**Figure 8:**
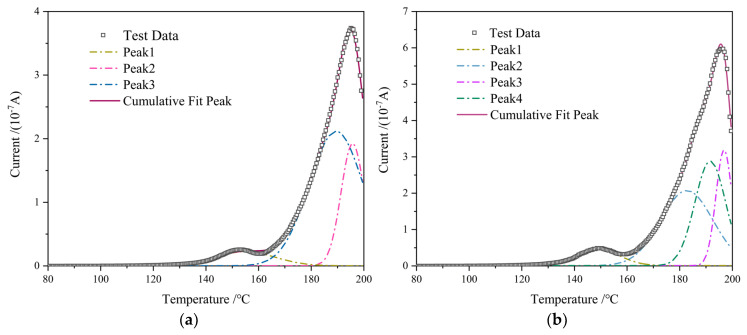
Peak fitting of thermal stimulated depolarization current curve of different samples. (**a**) Interface-free specimens; (**b**) interface specimens.

The TSDC curves of samples with and without interfaces measured in the experiment are shown in Figure 8. The main methods for analyzing TSDC curves include the initial rise method, half peak width method, and full curve calculation method [19]. The full curve calculation method has the advantages of a high degree of calculation accuracy and small error. This article used this method to calculate the trap energy level and trap charge quantity. Before calculating the trap parameters, the original TSDC curve was first divided into peaks, and then the individual peak parameters were calculated separately. The results are shown in Table 2, and the fitting degree of each peak in this paper is greater than 99%.

Reference [16] tested the dynamic mechanical properties of samples with or without interfaces. From the mechanical loss curve, it can be seen that the loss peak, which appears around 150 °C, is caused by the glass transition temperature of the epoxy resin. The high-temperature loss peak of samples with interfaces corresponds to a slightly lower temperature than those without interfaces, indicating that the introduction of interlayer interfaces reduces the glass transition temperature of epoxy resin materials. According to the temperature range of fitting peak 1 in the figure, it can be determined that peak 1 is caused by dipole polarization during the glass transition process, which is mainly caused by the main chain movement of epoxy molecules. The introduction of interlayer interfaces reduces the trap depth of peak 1. This is because the presence of interfaces can affect the formation of a three-dimensional network structure during epoxy curing, thereby affecting the degree of epoxy cross-linking. The chain segment movement of epoxy near the interface is easier, and the relaxation polarization process is more obvious, resulting in a decrease in trap depth compared to unbounded samples. When the temperature is higher than the glass transition temperature, the internal relaxation process of the resin becomes more complex. Peak 2 may be the electrode polarization of the epoxy resin, and the current peak amplitudes caused by the electrode polarization of the samples with or without an interface are close. This indicates that the influence of the interlayer interface on electrode polarization is relatively small, and the calculated trap energy level is also close. Peak 3 corresponds to the DC conductivity effect of epoxy resin at high temperatures. When the polarization voltage is applied, the carriers in the epoxy resin exhibit conductivity components in the broadband dielectric spectrum. As the polarization rapidly cools down, they are fixed in the epoxy resin and detach from the deep traps inside the material at high temperatures. 

Comparing the difference between the depolarization current peak and the trap energy level of the sample peak 3 with and without a boundary, it can be seen that the weak cross-linking region at the interface restricts the carrier movement. For samples containing interfaces, there is a hidden peak 4 above the glass transition temperature. At high temperatures, the conductivity values of the two samples show significant increases, and the interface polarization is obvious. In addition, the fitted peak has a high trap energy level and a large amount of trap charge, indicating that the introduction of interlayer interfaces brings a large number of deep traps. The introduction of deep traps is related to the cross-linking process in the interlayer interface region.

## 3. Analysis and Discussion

**Figure 9:**
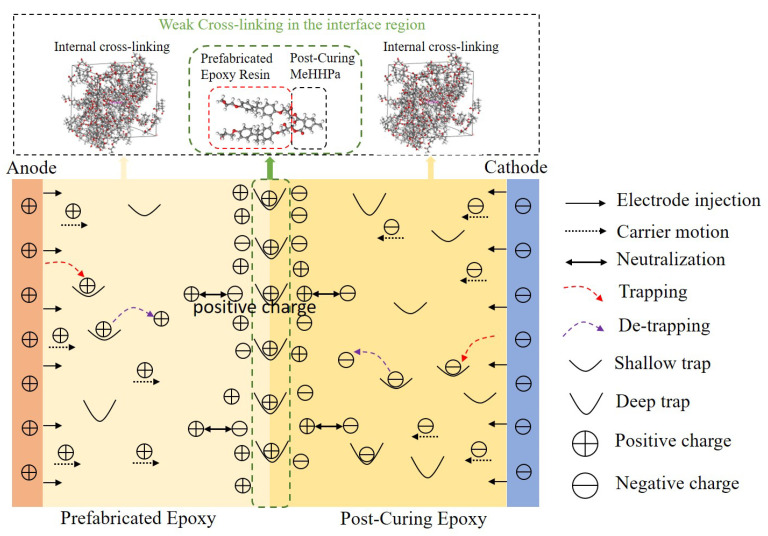
Schematic diagram of internal space charge transport in interlayer interface samples.

The epoxy resin in the prefabrication layer first forms a cross-linked network through the curing process, and then when the secondary pouring curing is carried out, the post-curing epoxy forms a cross-linked network. However, there are incomplete curing groups near the middle surface of the prefabrication layer (that is, the interlayer interface), such as hydroxyl groups and secondary hydroxyl groups generated by ring-opening reactions, and these groups will undergo cycloaddition with the epoxy groups in the post-curing epoxy. Therefore, the functional groups in the post-curing epoxy resin can move and react with the incompletely reacted functional groups in the prefabricated epoxy resin layer, resulting in a local cross-linking network at the interface. A schematic diagram of weak cross-linked and space charge transport is shown in Figure 9.

Considering that the prefabricated epoxy has already solidified and the material thickness is limited, the degree of epoxy body cross-linking is much greater than the weak cross-linking process at the interface, especially because as the temperature increases, the molecular chain segment activity in the weak cross-linking area at the interface becomes more active. When the curing process reaches a certain level, the molecules in the weakly cross-linked region will experience relaxation, tension, expansion, and contraction and form structures with discrete chemical functional groups. This leads to the change of energy levels and the generation of surface traps, thus introducing a deep trap here. In other areas where the epoxy is cross-linked internally and weakly at the interface, there are also epoxy groups that are not involved in the secondary curing reaction. These groups also appear near the interface. The chemical bond between the uncured epoxy resin and the anhydride breaks, forming holes and free radicals, and trap distribution is formed through redox. In addition, there may also be organic impurities, physical defects, and loose structures in the weakly cross-linked region which may also lead to the generation of deep traps. These defects can lead to changes in electron density in the region, forming electron dispersion or a local bandgap, thereby increasing the number of deep traps. Deep trap traps capture a large number of charge carriers, and there is charge accumulation near the interface.

Despite the existence of a weak cross-linking reaction, a large number of chemical bond and chain segments will “terminate” at the interface, and the surface states at the interface are significantly different from the body [20]. The surface state affects the spatial distribution and mobility of charges in materials, as well as changes in surface potential energy and energy system structure, thereby affecting the trap distribution. Overall, the trap characteristics and distribution mechanisms of interlayer interface structures formed through physical curing methods are influenced by various factors. When designing and preparing double-layer epoxy resin materials, it is necessary to pay attention to the weak cross-linking process at the interface to reduce the influence of deep traps.

In addition to the interlayer interface between epoxy and epoxy, the interface between the electrode and epoxy resin has a significant impact on the distribution of internal space charges [21,22,23,24], especially with the increase in charge carriers injected from the electrode–epoxy interface as the applied electric field increases. In this article, there is no significant charge accumulation near the electrode, which is mainly related to the contact state, work function, and interface potential barrier of the two materials. The impact mechanism of the charge injected into the electrode on the accumulation of interlayer interface charge and the deep traps introduced by weak cross-linking requires further research.

## 4. Materials and Methods

### 4.1. Sample Preparation

In this paper, a bisphenol A epoxy resin E39 produced by Nantong Xingchen Synthetic Materials Co., Ltd. in Nantong, China, was selected as the curing agent. Methyl hexahydrophthalic anhydride (MeHHPa), produced by Henan Puyang Huicheng Electronic Materials Co., Ltd. in Henan, China, was selected as the acid anhydride equivalent of 168.19 g/mol. Additionally, 2-ethyl-4 methyl imidazole (2,4-EMI), produced by Shanghai Chemical Industry Development Co., Ltd. in Shanghai, China, was selected as the accelerator. The formula system selected in this article was based on the content ratio of the epoxy resin, curing agent, and accelerator, which was 100:85.7:0.5. First, prepare the epoxy resin-impregnating solution. Pour the epoxy resin, curing agent, and accelerator into a beaker at the weight ratio of 100:65.5:0.5. Stir them evenly with a magnetic stirrer at room temperature to fully mix them. Then, place the beaker of the epoxy resin-impregnating solution into a vacuum oven for degassing. After degassing, allow it to stand for 20 min. Then pour the impregnating solution into the mold and degas again. After the bubbles disappear completely, cure it at 80 °C for 2 h, and cure it at 120 °C for 4 h and at 150 °C for 4 h. After the curing process is completed, waiting for the sample to gradually cool down, assemble the poured sample into the cleaned, new mold, and pour the pre-prepared epoxy resin-impregnation solution into the remaining space of the new mold. Perform degassing and solidification again. Finally, a bilayer epoxy resin sample with interlayer interfaces is obtained. When pouring the second layer of the epoxy resin-impregnation solution, it is necessary to ensure that the impregnation solution fully covers the remaining space in the mold to prevent the occurrence of air gaps at the interface. The presence of air gaps will greatly affect the material properties of the laminated epoxy resin. The first layer of pouring sample is named the prefabricated epoxy, and the second layer of pouring sample is named the post-curing epoxy.

### 4.2. PEA

**Figure 10:**
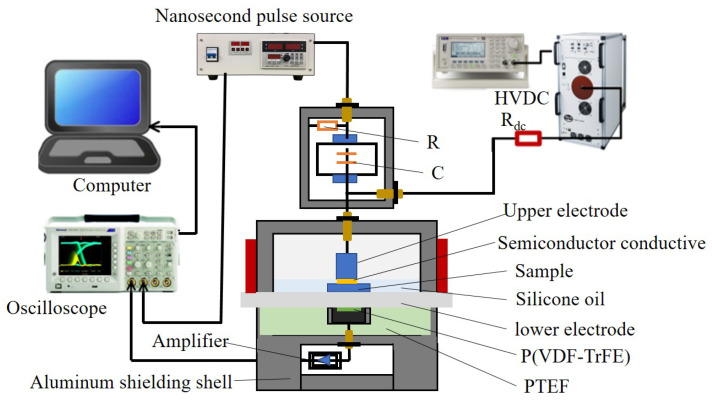
Space charge measurement system.

In this research, the high-temperature PEA method was used to measure the space charge distribution of the sample, as shown in Figure 10. This system includes piezoelectric sensors, R-C pulse coupling circuits, signal amplifiers, digital temperature controllers, DC high-voltage sources, nanosecond pulse sources, and other devices. Among them, the sensor uses a new type of high-Curie-temperature piezoelectric sensor, polyvinylidene fluoride trifluoroethylene (P (VDF TrFE)), with a maximum operating temperature of 120 °C, which is a significantly improved temperature resistance compared to regular PVDF. The signal amplifier was thermally isolated using polytetrafluoroethylene. The DC high-voltage source was matched with a power amplifier through an arbitrary waveform generator. Through programming, this voltage source system can also generate any waveform voltage, such as AC or AC and DC superimposed voltage. During the experiment, the sample was immersed in silicone oil to make the temperature uniform and stable and prevent the occurrence of surface discharge.

### 4.3. TSDC

The TSDC is widely used to measure parameter information such as trap energy level and trap density in insulating materials. This article first conducted a vacuum-drying treatment on the sample before the experiment to try to remove the internal moisture as much as possible. The TSDC test process involves placing the sample on the electrode and in a vacuum in a temperature-controlled chamber, heating it up to a polarization temperature of 160 °C, maintaining the temperature constant, and applying a polarization voltage to both ends of the sample, resulting in a polarization field strength of 0.3 kV/mm which lasts for 40 min. Then, quickly cool the sample to −120 °C, removing the depolarization voltage, and short circuit the two ends of the sample for 5 min. Finally, the temperature rises linearly at the rate of 3 °C/min, and the current in the external circuit, which is called the depolarization current, is measured with a Keithley 6517B Electrometer. The thermally stimulated depolarization current of the sample is shown in Formula 1. The amount of trapped charge can be calculated from the area surrounded by the TSDC curve and the X axis, and its expression is shown in Formula (2) [25]. The integrals are calculated using Gaussian quadrature and segmented cubic spline interpolation, respectively. The characteristic parameters of the TSDC separation peak can be obtained according to Formulas (3) and (4).

(3) $$I(T)=n_{0}s\exp(-\frac{E}{kT})\exp\left[-\frac{s}{\beta }\int_{T_{0}}^{T}e^{-\frac{E}{kT^{\prime }}}dT^{\prime }\right]$$

(4) $$Q=\frac{1}{\beta }\int_{T_{0}}^{T}I\left(T\right)dT^{\prime }$$

where *n*_0_ is the initial trap charge concentration; *s* is the frequency factor, representing the probability of thermally excited carriers being neutralized or recaptured; *E* is the trap energy level; *Q* is trap charge quantity; *K* is the Boltzmann constant; *T* is the absolute temperature with unit of K; *β* is the heating rate; *T*_0_ is the initial temperature during the heating process.

## 5. Conclusions

In this research, the PEA and TSDC methods were used for a cooperative analysis of the space charge accumulation characteristics of the sample and the influence of a weak cross-linking process on the trap characteristics at the interface of a double-layer epoxy resin.

Although the same materials were used on both sides of the interface, interface polarization still occurred at the interface under high temperature and high field conditions. This interfacial polarization was mainly caused by weak cross-linking reactions at the interface. The functional groups in the post-curing epoxy resin can move and react with some of the reactive functional groups in the prefabricated epoxy resin, forming a local sparse cross-linking network at the interface. The smaller cross-linking density makes the molecular chain segments at the interface more active and introduces deep traps at the interface, thereby causing the process of interface charge accumulation.

Experimental testing shows that the ability of the interface charges to cause a field strength distortion decreases with the increase in the applied field strength. The distortion effect of the space charge on the electric field reaches its maximum at 80 °C and 10 kV/mm, with a maximum distortion rate of 15.8%.

## Figures and Tables

**Table 1:** Distortion factor of field strength at different temperatures and field strengths.

Temperature/°C	Field Strength *E*_p_/kV·mm^−1^	Maximum Field Strength *E*_max_/kV·mm^−1^	Field Distortion Factor *F*_d_/%
80	10	11.58	15.8
	20	20.78	3.9
100	10	11.36	13.6
	20	21.15	5.8

**Table 2:** Different fitting peak trap parameters.

Peak (Sample) Type		Trap Energy Level (eV)	Trap Charge Quantity (μC)
Peak 1	Interface-free specimens	1.13	0.212
	Specimens with interface	0.89	0.308
Peak 2	Interface-free specimens	1.27	1.515
	Specimens with interface	1.29	1.635
Peak 3	Interface-free specimens	2.20	0.566
	Specimens with interface	2.31	0.659
Peak 4	Specimens with interface	2.06	1.279

## Data Availability

Where data is unavailable due to privacy or ethical restrictions.
